# Bridged EGFET Design for the Rapid Screening of Sorbents as Sensitisers in Water-Pollution Sensors

**DOI:** 10.3390/s23177554

**Published:** 2023-08-31

**Authors:** Hadi Rasam AlQahtani, Abdel-Basit M. Al-Odayni, Yusif Alhamed, Martin Grell

**Affiliations:** 1 Department of Physics and Astronomy, College of Science, King Saud University, Riyadh 11451, Saudi Arabia; 2 Engineer Abdullah Bugshan Research Chair for Dental and Oral Rehabilitation, College of Dentistry, King Saud University, Riyadh 11451, Saudi Arabia; 3 Llyfrgell Bangor, Ffordd Gwynedd, Bangor LL57 1DT, UK; martin@spinne.plus.com

**Keywords:** EGFET, water monitoring, Puromet, mercury pollution, Langmuir–Freundlich

## Abstract

We further simplify the most ‘user-friendly’ potentiometric sensor for waterborne analytes, the ‘extended-gate field effect transistor’ (EGFET). This is accomplished using a ‘bridge’ design, that links two separate water pools, a ‘control gate’ (CG) pool and a ‘floating gate’ (FG) pool, by a bridge filled with agar-agar hydrogel. We show electric communication between electrodes in the pools across the gel bridge to the gate of an LND150 FET. When loading the gel bridge with a sorbent that is known to act as a sensitiser for Cu^2+^ water pollution, namely, the ion exchanging zeolite ‘clinoptilolite’, the bridged EGFET acts as a potentiometric sensor to waterborne Cu^2+^. We then introduce novel sensitisers into the gel bridge, the commercially available resins Puromet^TM^ MTS9140 and MTS9200, which are sorbents for the extraction of mercury (Hg^2+^) pollution from water. We find a response of the bridged EGFET to Hg^2+^ water pollution, setting a template for the rapid screening of ion exchange resins that are readily available for a wide range of harmful (or precious) metal ions. We fit the potentiometric sensor response vs. pollutant concentration characteristics to the Langmuir–Freundlich (LF) model which is discussed in context with other ion-sensor characteristics.

## 1. Introduction

Clean drinking water is essential for human life. Therefore, the World Health Organisation (WHO), as well as many national and international bodies, set maximum acceptable ‘potability’ levels of pollutants for water to be fit for human consumption (‘potable’) [1]. To monitor potability, we require analytical technologies and/or sensor devices that selectively detect and quantify target analytes like pollutants and pathogens in an aqueous medium, herein called ‘water’ for short. An important ‘family’ of devices for this purpose are potentiometric sensors based on field effect transistors (FETs) as transducers. Transistor-based sensors work on a far smaller experimental footprint than traditional analytical methods such as mass spectroscopy, e.g., Allibone et al. [2]. The discipline goes back to the invention of the ‘ion-sensitive field effect transistor’ (ISFET) by Bergveld in 1970 [3]. The ISFET is a field effect transistor immersed into the water sample under test, and functionalised with selective ‘sensitisers’ (also known as ‘receptors’) that bind to the target analyte, leading to an interface potential that shifts the ISFET’s transfer characteristics along the gate voltage axis. After prior calibration, the presence and magnitude of such a shift can be related to the presence and concentration of target analyte in water. Many research groups have since used field effect transistors to target a variety of pollutants and biomedical analytes, pesticides [4], hormones [5], viruses [6,7,8], bacteria [9], and cancer markers [10]. Transistor sensors for biomedical analytes have been named ‘BioFETs’; a recent review is, e.g., [11]. Nevertheless, to date, ions remain an important group of target analytes for all types of field effect sensors, e.g., [12,13,14,15,16,17]. Mao et al. [18] reviewed recent advances in field effect sensors for the detection of heavy metal pollution in water, which is a serious concern due to the strong toxicity of many heavy metals. Table 1 shows an overview of field effect sensors for mercury (Hg^2+^) pollution reviewed by Mao et al., with the addition of Sukesan et al. [19]. Reference [1] sets a potability limit for Hg^2+^ of 29.9 nM; several national regulators demand lower potability limits in the order ~10 nM. Most of the sensors listed in Table 1 show a limit-of-detection (LoD) < potability. We note that the EGFET is the most ‘user-friendly’ field effect transducer, as it avoids chemical modification of the transistor itself as in the ISFET, and manufacture of the transistor substrate (source/drain contacts, semiconducting channel) by the experimenters, which is required for the WGTFT. But while EGFETs are often used for the sensing of pH, i.e., H_3_O^+^ ions, e.g., Al-Hardan et al. [20], only one EGFET sensor, that of Sukesan et al. [19], is included in Table 1. The review by Mao et al. [18] even missed the work of Sukesan et al., listing only ISFETs and WGTFTs.

We believe that the EGFET has been neglected in the recent development of field effect sensors, maybe due to the novelty of the closely related WGTFT transducer concept. A focus on the transducer concept, however novel, distracts attention from the most important challenge in FET sensor technology, namely, the identification and immobilisation of suitable sensitisers. To redress this imbalance, we here report a novel ‘bridged’ EGFET design for the rapid screening of potential sensitisers with a minimum of cost and effort to be expended on the transducer. As convenient examples, we chose Cu^2+^ and Hg^2+^ as analytes to develop our transducer concept, as these both are important water pollutants (*cf.* Table 1) with readily available sorbents known for their extraction that we can test as sensitisers. We first confirm a response of a bridged EGFET to Cu^2+^, using a sensitiser known from WGTFT studies, i.e., Alqahtani et al. [16]. Then, we apply the bridged EGFET concept for a successful test of commercial mercury sorbents resins, MTS9140 and MTS9200, supplied by Purolite under the Puromet^TM^ brand [31], as sensitisers for mercury detection. This sets a template for screening the entire catalogue of Purolite sorbents for water purification as potential sensitisers in potentiometric sensors.

## 2. Experimental Section

### 2.1. ‘Bridged’ EGFET Desgin

**Figure 1:**
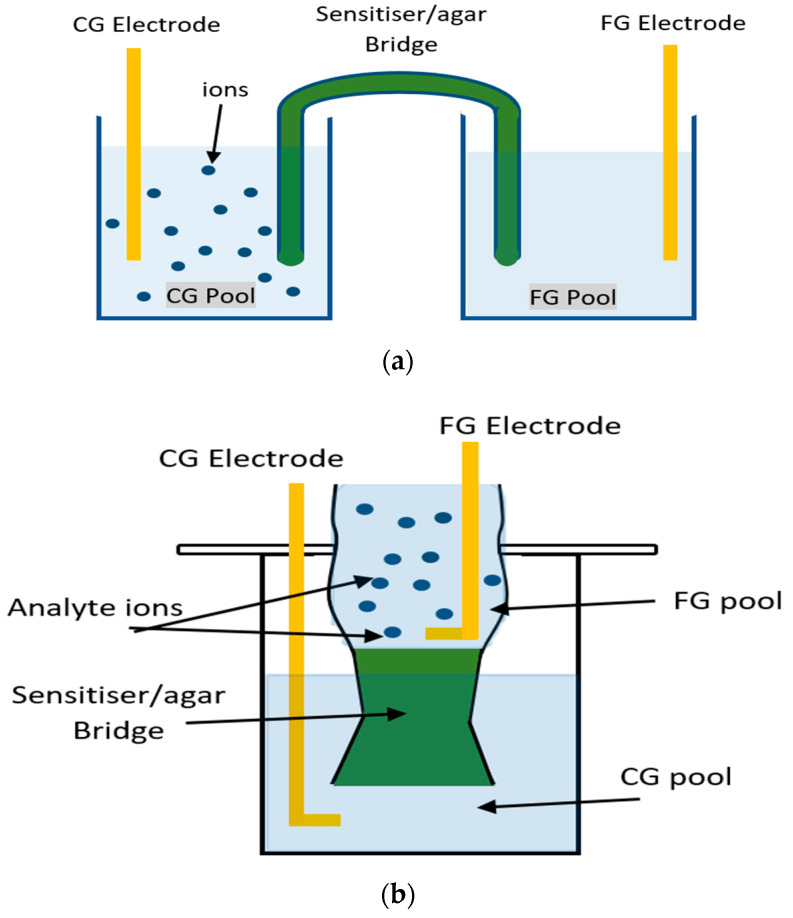
‘Bridged’ EGFET designs. (**a**). U-tube bridge, (**b**). funnel bridge. CG and FG communicate via a pair of interfacial electric double layers (EDLs) (only) after inserting a ‘bridge’ (an upturned U-tube (**top**)) or a ‘funnel’ (**bottom**) to connect the two pools. The bridge is filled with agar-agar hydrogel and entrapped sensitiser. CG is connected to a source-measure unit (SMU), sweeping CG voltage V_CG_. FG is connected to the gate of an LND150 FET, i.e., FG is the ‘extended gate’. For experiments with the U- tube bridge, we titrated analyte into the CG pool (CG pool = ‘sample pool’), Figure 3a–c. For experiments with the ‘funnel’, we titrated analyte into the FG pool (FG pool = ‘sample pool’), Figures 2 and 4.

In a conventional EGFET (e.g., Könemund et al. [9], Al-Hardan et al. [20]), the control gate (CG), i.e., the electrode connected to the gate voltage source, and the ‘floating gate’ (FG), i.e., the electrode connected to the FET gate terminal, are immersed in the same pool. Here, we redesigned the EGFET by separating CG and FG into two separate pools, connected by a ‘bridge’. CG and FG electrodes were gold rods and were both immersed into separate pools of 100 mL volume each, but pools were only filled with water to a level of 50 mL to allow later titration of analyte. Our ‘bridged’ EGFET designs are illustrated in Figure 1. 

### 2.2. Processing and Electrical Characterisation

As ‘water’ we used locally drawn tap water rather than DI water to provide a realistic background ‘cocktail’ of dissolved common salts. Also, local tap water has a pH ~ 7 (near neutral), for which commercial ion exchange resins for drinking water treatment are designed [31]. Ion extraction may be affected by ‘extreme’ pH, but that is more pertinent to the treatment of industrial wastewater effluent, e.g., Sharrad et al. [32]. We bridged the two pools either by U-tube or a ‘funnel’ (Figure 1). As a U-tube ‘bridge’, we filled a U-shaped glass tube (7 cm length, 1 cm diameter, volume 13.5 mL) with agar-agar hydrogel, mixed with the respective sensitiser. Alternatively, we used a glass funnel to hold agar gel. The funnel’s bottom end was necked and sloped up and down (*cf.* Figure 1b) at a small angle in order to keep the gel fixed in position. We filled between 10 and 15 mL of sensitised hot agar solution into the funnel, allowed it to cool and set, then added 20 mL (half the funnel’s total capacity) of water above the set gel. Agar-agar powder (Probios Organic Seaweed Agar) was sourced from True-Beauty_KSA on Amazon Marketplace. Sensitised hydrogel was prepared by dissolving agar powder in water, bringing the solution near 80 °C, adding powdered sensitiser, mixing, filling into the bridge, and allowing to cool. As temperature drops below 40 °C, agar-agar forms a soft but solid hydrogel [33]. The powdered sensitiser becomes entrapped in the agar gel, similar to that reported, e.g., by Prakash et al. [34], avoiding the difficulties of covalent immobilisation, as reviewed by Duval et al. [35]. After the sensitiser/agar mix had cooled and gelled, the U-tube was upturned, with one leg of the U-tube immersed in CG pool and the other in FG pool. The upturned U-tube forms the ‘bridge’ that allows electric communication between CG and FG. Alternatively, the funnel was inserted into a glass beaker filled with ~100mL of tap water. The FG electrode was inserted into the water pool at the top of the funnel, and the CG electrode was inserted into the glass beaker.

As a sensitiser for Cu^2+^ ions, we used a fine powder of the zeolite clinoptilolite, grain size < 40 μm, as received from ‘DC Minerals’ at a weigh-in of about 0.1 g/mL hot agar solution before filling the funnel. Clinoptilolite is a zeolite (aluminosilicate) with a unit cell defined by a Si:Al:O ratio of 30:6:72. The 6 ‘missing’ valencies resulting from replacing 6 Si(IV) with Al(III) are compensated by incorporating a mix of common ions like Na^+^, K^+^, and Ca^2+^. However, these are not bonded very tightly and are readily exchanged for Cu^2+^ or Pb^2+^ when the latter become available. Clinoptilolite is therefore used as a sorbent for the purification of water contaminated with Cu^2+^ and/or Pb^2+^ (Perić et al. [36]). Alqahtani et al. [16] demonstrated that the sorbent clinoptilolite also acts as a sensitiser for lead and copper within a WGTFT sensor platform. As a sensitiser for Hg^2+^, we used Purolite resins MTS9140 and MTS9200 [31], sourced from Purolite Middle East, Amman, Jordan. MTS9140 and MTS9200 are ion exchangers for waterborne mercury (Hg^2+^), *cf*. Discussion in part 4. These resins can extract more than their own weight of mercury from polluted water [31] before becoming exhausted, which far exceeds the amount of mercury they will be exposed to in our experiments. Purolite resins have not previously been used as sensitisers. We ground both resins and added them to a hot agar solution, either separately or mixed in a 1:1 ratio by weight. Type of bridge, type and concentrations of sensitiser, and concentration of agar powder are summarised in Table 2.

**Table 2:** Type of bridge and concentrations of all ingredients for the agar gel bridges used here. All gels were prepared in tap water. The final column refers to the figure in part 3 for which the respective bridge was used.

Bridge Type	Sensitiser	Sensitiser Concentration	Agar Concentration	Fig.
Funnel	Clinoptilolite	150 mg/mL	20 mg/mL	Figure 2
U-shape	MTS 9140/MTS 9200 1:1	200 mg/mL	40 mg/mL	Figure 3a
U-shape	MTS 9140	200 mg/mL	40 mg/mL	Figure 3b
U-shape	MTS 9200	200 mg/mL	40 mg/mL	Figure 3c
Funnel	MTS 9140/MTS 9200 1:1	100 mg/mL	20 mg/mL	Figure 4

For Cu^2+^ as the analyte, we prepared a 10 mM stock solution of Cu^2+^ from CuSO_4_.5H_2_O. For Hg^2+^ as the analyte, we prepared a 5 μM stock solution of Hg^2+^ from Hg(II)Nitrate monohydrate (Hg(NO_3_)2·H_2_O) sourced from Sigma (83381-50G). As a field effect transistor, we used the LND150 ‘normally on’ (depletion-mode) n-channel FET [37], a common choice for EGFET work, e.g., [9,19]. LND150 gate was extended by connecting to the FG contact. Electrical characterisation was with a Keithley 2634B dual channel source-measure unit (SMU). To record saturated transfer characteristics, the LND150 source was grounded, +10 V were applied to the drain, and control gate (CG) voltage was swept through a closed voltage cycle, 0V → +1V → −1V → 0V, in steps of 50 mV for Cu^2+^ sensing and 20 mV for Hg^2+^ sensing. After each CG voltage step, we allowed 1.5 s for EDLs to build up in full and recorded the resulting FET drain current. We recorded the first saturated transfer with both the FG and CG pool filled with (tap) water only. Then, we titrated small aliquots of analyte stock solution from a graded pipette into either the CG (U-tube) or the FG (funnel) pool to raise analyte concentration step by step. Work was carried out in a climatised lab at 21 °C to avoid possible influences of temperature variations. For each increased analyte concentration, we then recorded the LND150 saturated transfer again. The sets of transfers under increasing analyte concentration are shown and analysed in ‘Results and Discussion’.

## 3. Results

### 3.1. Sensing Cu^2+^ with a Funnel-Bridged EGFET Sensitised with Zeolite Sorbent

**Figure 2:**
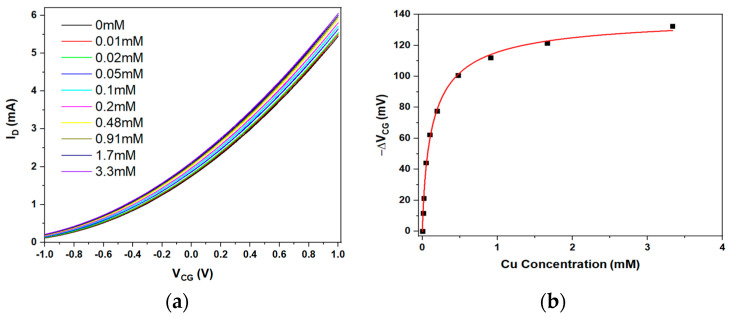
(**a**). LND150 saturated transfer characteristics in funnel-bridged EGFET configuration. The bridge was sensitised with clinoptilolite, and we titrated increasing concentration of Cu^2+^ into the FG pool. (**b**). Quantitative evaluation of control gate (CG) voltage shift vs. Cu^2+^ concentration, c, using Equation (1). The red curve is a fit to Equation (2).

We bridge the CG/FG pair of pools with a funnel bridge sensitised with powdered ‘clinoptilolite’, prepared as described in Section 2. In Figure 2a, we show the LND150 transfer characteristics vs. CG voltage when titrating increasing concentrations of Cu^2+^ into the CG pool. 

Figure 2a shows that the saturated drain current in the LND150 clearly increases with increasing CG voltage, confirming electric communication between CG and FG across the bridge. Further, while transfer characteristics retain their shape, they clearly shift to more negative CG voltage with titration of increasing concentration, c, of Cu^2+^. Hence, the use of clinoptilolite as a sensitiser for Cu^2+^ translates from the WGTFT (Alqahtani et al. [16]) to the EGFET platform. As is common for EGFETs (e.g., Al-Hardan et al. [20]), concentration-dependent CG voltage shift ΔV_CG_(c) is quantified by evaluating V_CG_ required to achieve a particular (somewhat arbitrarily chosen) drain current, here I_D_ = 1.75 mA, as in Equation (1):

ΔV_CG_(c) = |V_CG_(I_D_ = 1.75 mA, c) − V_CG_(I_D_ = 1.75 mA, c = 0)| (1)

**Figure 3:**
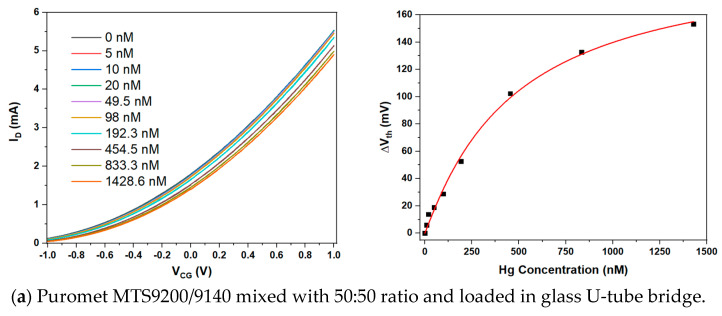

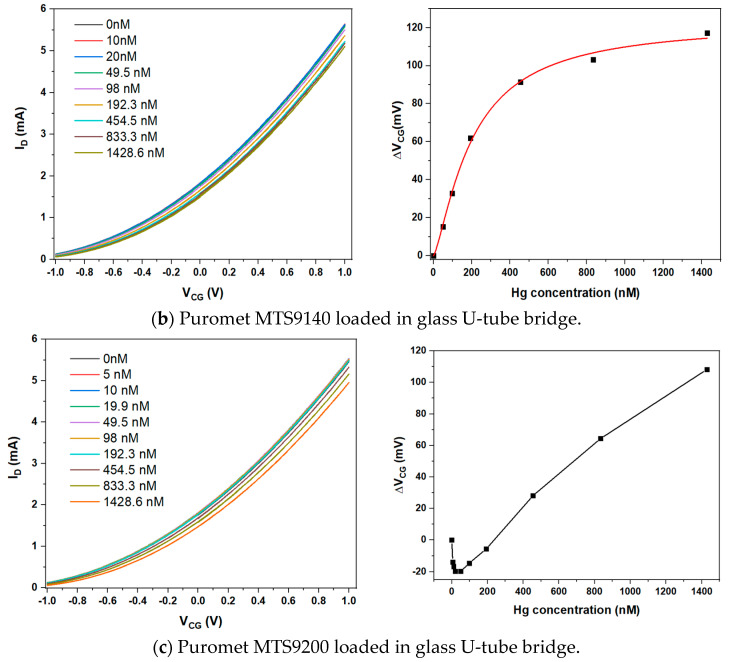
LND150 saturated transfer characteristics in a bridged EGFET configuration sensitised with Puromet mercury sorbents under titration of increasing concentration, c, of Hg^2+^ into the into the CG pool. Also shown, quantitative evaluation of resulting CG voltage shift using Equation (1). (**a**): Bridge sensitised with mixed MTS9140 and MTS9200 resin, and quantitative response characteristics (red curve) fitted to Equation (2). (**b**): Bridge sensitised with MTS9140 only, and quantitative response characteristics (red curve) fitted to Equation (2). (**c**): Bridge sensitised with MTS9200 only, and quantitative response characteristics, without fit.

Note we define ΔV_CG_(c) as a modulus, i.e., we always report ΔV_CG_(c) as positive, although its sign is found to be different in different experimental setups. As an exception, Figure 3c was not evaluated as a modulus, as ΔV_CG_(c) changes sign. The sign of ΔV_CG_ is discussed at the top of paragraph 4. 

The CG shift of ΔV_CG_(c) as evaluated from Figure 2a is plotted in Figure 2b. For quantitative analysis of response characteristics, we used the same model as Alqahtani et al. [16], based on the Langmuir–Freundlich (LF) adsorption isotherm. Equation (2):

(2) $$\Delta V_{\mathrm{CG}}(c)=\Delta V_{\mathrm{sat}}\;\frac{{{{\left(kc\right)}^{\beta }}^{\;}}^{\;}}{1+{{{\left(kc\right)}^{\beta }}^{\;}}^{\;}}$$

where ΔV_sat_ is the CG shift in the limit of large analyte concentration c, k is a constant with units of inverse concentration, and β is a dimensionless exponent. ΔV_CG_ reaches ½ ΔV_sat_ for c_1/2_ = 1/k. A good fit is obtained, and fit parameters are summarised in Table 3 below.

### 3.2. Sensing Hg^2+^ with a U-Tube Bridged EGFET Sensitised with Purolite Sorbent Resins

Purolite supplies a range of functionalised resins based on a crosslinked polystyrene matrix as sorbents for the extraction of pollutants from drinking water under the Puromet^TM^ brand [31]. To quickly assess the potential of these sorbents in potentiometric sensors, we introduced Puromet mercury (Hg^2+^) sorbents MTS9140 and MTS9200 into the EGFET platform via U-tube ‘bridges’, as described in the Experimental Section. Then, we recorded transfer characteristics under titration of increasing concentrations of Hg^2+^ into the CG pool. The results are shown in Figure 3, Figure 3a: Bridge contains a mix of MTS9140 and MTS9200, Figure 3b: Bridge contains MTS9140 only, and Figure 3c: Bridge contains MTS9200 only.

Again, we see a clear response of the bridged EGFET sensor to analytes, in this case Hg^2+^. While Puromet resins are chemically very different from clinoptilolite (functionalised organic resin vs. inorganic crystal), both extract their ‘target’ heavy metal ion from water by exchanging it for other, harmless ions. In the case of Puromet resins, Hg^2+^ will be complexed by the resin’s functional thiourea (MTS9140) or isothiouronium (MTS9200) groups under exchange for 2H^+^ ions. Quantitative response characteristics for Figure 3a (MTS9140 + MTS9200) and Figure 3b (MTS9200) are again fitted well by the LF approach (Equation (2)). Fit parameters are listed in Table 3. The response characteristics in Figure 3c (MTS9200) is unusual as it changes sign, with a minimum near the WHO potability. No model was fitted to this response characteristic.

### 3.3. Sensing Hg^2+^ with a Funnel-Bridged EGFET Sensitised with Purolite Sorbent Resins

**Figure 4:**
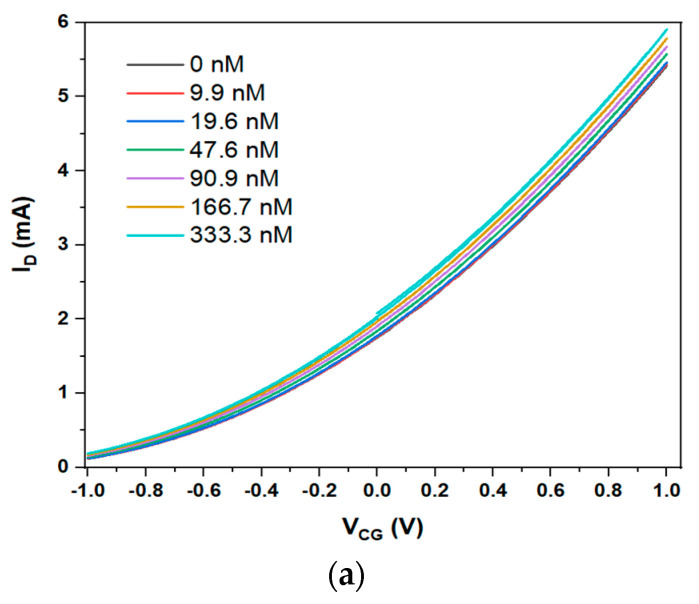

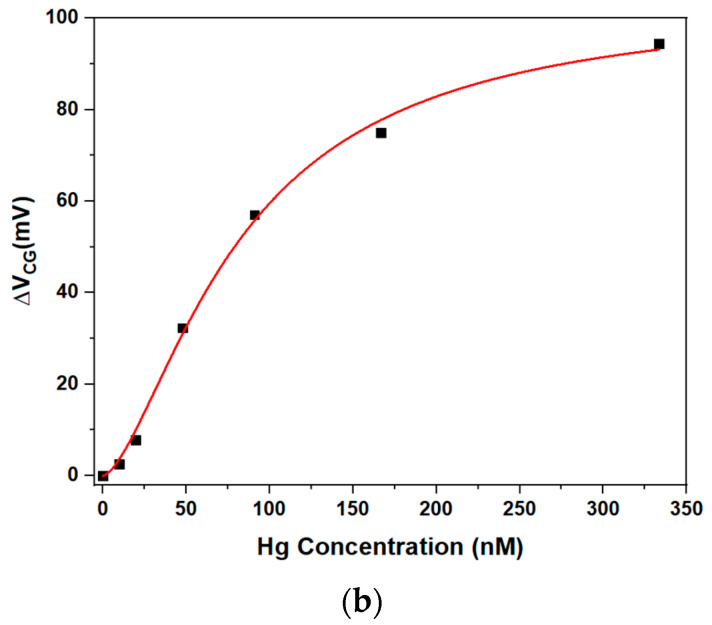
(**a**) LND150 saturated transfer characteristics (VD = 10 V) in a ‘funnel’ EGFET configuration sensitised with mixed Puromet MTS 9140 + 9200 mercury sorbents under titration of increasing concentration, c, of Hg^2+^ into the FG pool. (**b**) Quantitative evaluation of resulting CG voltage shift and the red curve is a fit to Equation (2).

We repeated the successful Hg^2+^ sensing experiment, Figure 3a, with the same mixed Puromet resins, but in a different EGFET sensor design, i.e., the ‘funnel’ described in the Experimental Section. The results are in Figure 4.

The parameters for fitting Equation (2) to response characteristics are summarised above in Table 3. The limit-of-detection (LoD) is evaluated as described in the Supplementary Materials.

## 4. Discussion

The origin of ΔV_CG_, and its sign, are understood by assuming that ion exchange between sensitiser in the gel and analyte cation diffusing into the gel from the titration pool leads to a dipole moment (→) at the interface (∥) between (water + cation) and sensitised gel. By our definition (Equation (1)), ΔV_CG_ is always reported as positive to simplify presentation. However, the sign of ΔV_CG_ was negative when analyte was titrated into the FG pool (Figure 1 and Figure 4), but positive when analyte was titrated into the CG pool (Figure 3a,b). The dipole moment will face into the opposite direction with respect to V_CG_ when the analyte cation is present in the CG pool vs. in the FG pool:

CG electrode ‖ (water + cation) ⇻ gel ‖ water ‖ FG electrode

vs.

CG electrode ‖ water ‖ gel ⇺ (water + cation) ‖ FG electrode

Quantitatively, we find that our response characteristics of ΔV_CG_ vs. analyte concentration c are fitted well by the Langmuir–Freundlich (LF) model (Equation (2)). LF characteristics differ significantly from a previous report on a mercury-sensitive EGFET by Sukesan et al. [19]. Their response characteristics follow a refinement of the Nernst equation known as the ‘Nikolsky–Eisenman’ (NE) law, shown in Equation (3):

(3) $$\Delta V_{\mathrm{CG}}(c)=\frac{59mV}{z}\;\log\left(\frac{c}{c_{st}}+1\right)$$

where z is the valency of the extracted ion (z = 2 for mercury) and c_st_ is a concentration typical of a particular sensitiser, and somewhat dependent on interferants. At concentrations c >> c_st_, Equation (3) is virtually identical to the classic Nernst law, but at c < c_st_, Equation (3) flatlines, avoiding the unrealistic Nernstian divergence for c → 0. c_st_ sets a limit of detection (LoD) for an NE response. Remarkably, the NE law is independent of the weight-loading of the sensitiser in the membrane, and the response of different sensitisers differs only by the magnitude of c_st_. The NE response is observed for sensitisers that extract ions from water without ion exchange and therefore build up electric charge in the sensitised membrane. This is typical for organic macrocycles like calixarenes, cyclodextrins, or crown ethers, e.g., [12,14,38,39]. Similarly, Sukesan et al. [19] used the diazo crown ether ‘mercury ionophore I’ [40] which traps mercury ions in its central cavity without ion exchange. 

However, ion sorbents for the quantitative extraction (purification) of water from toxic ions, like clinoptilolite or Purolite resins, always have to be ion exchangers. This is to avoid the charging of sorbent grains, which would repel further sorbate ions and severely limit the uptake of harmful ions by the sorbent. This leads to different response characteristics for potentiometric sensors using such ion exchanging sensitisers, e.g., [15,16,17]. These often follow a Langmuir- or LF-type response characteristic (Equation (2)). Similarly, potentiometric biosensors (‘BioFETs’) often respond following the ‘Hill equation’ [41], e.g., Zhou et al. [10], which is mathematically equivalent to the LF law (see Alqahtani et al. [8], Section 4.3). The choice of transducer (WGTFT, EGFET, ISFET) is immaterial for the type of response characteristic. Consequently, we here also observe LF characteristics.

While the NE response characteristic Equation (3) for sensitisers without ion exchange is fully understood theoretically, the use of the LF response model (Equation (2)) is partly empirical. For biosensors, β is interpreted in terms of ‘cooperativity’, i.e., interactions between neighbouring sorbtion sites. The Langmuir model assumes the absence of such interactions, which leads to β = 1, in which case Equation (2) reproduces the classic Langmuir adsorption isotherm. Within Langmuir theory, the parameter k has a clearly defined meaning, namely, the association constant of the sorbent/analyte complex. However, k from fitting sensor characteristics can be orders of magnitude larger than the k for the same sorbent when evaluated from quantitative sorbtion studies [17]. Also, when clinoptilolite was used in a plasticised PVC membrane [16], k for the sorption of Cu^2+^ was found ≈36 times larger than here (*cf.* Table 3, Figure 2). β and ΔV_sat_ were significantly different as well. Further, the response in Figure 4 is different from that in Figure 3a, albeit using the same mix of sensitisers, but with a different ‘bridge’. Alghamdi et al. [15] found sensitiser particle size and weight loading in the membrane to be influential as well. Also, EGFET response amplification has been reached by tuning the aspect ratio (relative area) of CG (large) w.r.t. FG (small) [42]. This encourages further systematic experimentation to improve sensitivity and LoD, which is not possible for NE sensors.

LF and NE characteristics complement each other, resulting in sensors with different relative merits. The NE-type mercury-sensitive EGFET by Sukesan et al. [19] achieves a very low limit-of-detection (LoD) of c_st_ ≈ 0.3 pM, and a dynamic range (the range from LoD to saturation) of seven orders of magnitude. NE sensors with such low c_st_ (i.e., LoD) can be useful, e.g., for prospecting for metal deposits upstream. However, the LoD is far lower than required to assess potability w.r.t. mercury, *cf.* WHO potability c_Pot_ = 29.9 nM [1]. Hence, the sensor in [19] covers four orders of magnitude of mercury ‘pollution’ that is rather harmless. Instead of ultra-low LoD, the decision to accept or reject water for potability requires good resolution (ability to detect small differences) at c ≈ c_Pot_, which is quantified by the sensor’s sensitivity, S, defined as the derivative of ΔV_CG_(c) with respect to c, shown in Equation (4a):

S(c) = dΔV_CG_(c)/dc (4a)

As both NE and LF law are non-linear, S depends on c for both. The logarithmic character of the Nernst law (and NE law for c >> c_st_) leads to:

(4b) $$S_{N}(c)=\frac{59\;mV}{2.303z}\;\frac{1}{c}$$

For z = 2 as for Hg^2+^, S = 12.8 mV/c for z = 2 (divalent ions) as long as c >> c_st_, i.e., S, strongly drops with increasing c. At c_Pot_ = 29.9 nM for mercury, a device with NE response as in [19] has S_N_(c_Pot_) = 0.43 mV/nM. As Nernstian sensitivity is independent of the type and concentration of the sensitiser, this cannot be improved.

The LF response law we observed here also somewhat loses S with increasing c, but up to c ≈ 1/k this loss is moderate. For c << c_1/2_ = 1/k, the LF law is approximated by the Freundlich isotherm, ignoring the denominator in Equation (2). The sensitivity of the Freundlich law (for β ≠ 1) is given by:

(4c) $$S_{F}(c)=\beta k^{\beta }\Delta {{V_{sat}}_{\;}c^{\beta -1}}_{\;}=\frac{\beta }{c}\Delta V_{\mathrm{CG}}(c)$$

Our example in Figure 4, with the parameters from Table 3, gives a sensitivity of 1.13 mV/nM at c_Pot_ for mercury. Hence, despite a much higher LoD, an LF response sensor can have better resolution at c_Pot_. Unlike for a Nernstian sensor, resolution can be further improved in principle, for example, by increasing ΔV_sat_.

## 5. Conclusions

We further simplify the most ‘user-friendly’ potentiometric field effect sensor, the extended-gate field effect transistor (EGFET). This is by introducing a ‘bridged’ EGFET design, where we link two separate water pools, a ‘control gate’ pool and a ‘floating gate’ pool, by a ‘bridge’ filled with agar-agar hydrogel similar to an electrochemical salt bridge. Our design relies on stock components and widely accessible, non-toxic chemicals only. We show the two pools communicate electrically across the gel bridge. We worked with two bridge designs (‘funnel’ and ‘U-tube’) which give very similar limits-of-detection (*cf.* Table 3, Figure 3a vs. Figure 4). However, we find the U-tube design easier to handle and therefore recommend it for future work. 

The simple transducer design allows one to focus on the key challenge in sensor technology, the identification and evaluation of new sensitisers. We immobilise sensitisers by physical entrapment in the bridge’s hydrogel, avoiding the significant difficulties associated with covalent immobilisation [35]. The sensitiser can easily be changed within the otherwise same transducer platform. 

We first demonstrate the working of the ‘bridged’ EGFET concept with a sensitiser known to respond to Cu^2+^ in a water-gate thin film transistor (WGTFT), namely, the zeolite ion exchanger ‘clinoptilolite’ [16]. Then, we tap into a large sensitiser resource that is not yet explored. The Purolite company supplies a wide range of functionalised resins for the extraction of ionic pollutants from drinking water under the Puromet^TM^ brand [31]. We here show that the mercury sorbents MTS9140 and MTS9200 also act as sensitisers for the same pollutant in our bridged EGFET sensor design. This sets a template for screening the entire catalogue of Purolite sorbents for water purification as potential sensitisers, covering a wide range of harmful (and/or precious) metal ions such as antimony, bismuth, chromium(III), cobalt, copper, gallium, gold, nickel, rhenium, uranium, and many more.

Response characteristics are fitted well by a Langmuir–Freundlich (LF) response law, which is mathematically equivalent to the Hill equation that often quantifies potentiometric biosensors. This agrees with earlier work using ion-exchanging sensitisers and contrasts with the Nernst or Nikolsky–Eisenmann response law that is found with sensitisers that extract ions without exchange. We demonstrate a limit-of-detection below the WHO potability limit for mercury and larger than Nernstian sensitivity at the potability limit. Unlike for the Nernst law, the characteristic parameters for the LF response law are not theoretically limited, which encourages systematic experiments to amplify response.

## Figures and Tables

**Table 1:** Overview of FET sensors for Hg^2+^.

Ref.	Semiconductor	LoD	Type	Response	Functionalisation/Sensitiser
[21]	Si NW	100 nM	ISFET	Nernst	3-mercaptopropyl triethoxysilane
[22]	Graphene	556 μM	ISFET	NA	1-octanethiol
[23]	red. GO	≈1 nM	WGTFT	Langmuir	Protein
[24]	GO	1.2 μM	ISFET	Nernst	Tailored nucleic acid
[25]	Graphene	10 pM	WGTFT	Nernst	Aptamer
[26]	red. GO	10 pM	WGTFT	Nernst	Polyfuran
[27]	red. GO	1 nM	ISFET	NA	DNA
[28]	MoS_2_	30 pM	ISFET	Nernst	Innate (S in MoS_2_)
[29]	Graphene	5.6 nM	WGTFT	Langmuir	Innate Ionophore
[30]	MoS_2/_Au NPs	100 pM	ISFET	Langmuir	DNA
[19]	LND150	≈0.3 pM	EGFET	Nernst	Diaza crown ether

Overview of Hg^2+^-sensitive FET devices. Semiconductor: Si NW: Silicon nanowires. (red.) GO: (reduced) Graphene oxide. Au NPs: Gold nanoparticles. LND150: Commercial depletion-mode n-channel FET. LoD: Limit-of-detection. Type: ISFET: Ion-selective field effect transistor. WGTFT: water-gate thin film transistor. EGFET: extended-gate field effect transistor. Response characteristics: Langmuir, follows Langmuir or Langmuir–Freundlich (LF) type response law. Equation (2). Nernst, follows Nernst or Nikolsky–Eisenman (NE, modified Nernst) response law, Equation (3). The assignment is not always made in the original literature and was added here by the present authors. NA: Quantitative response characteristics were not clearly reported. Functionalisation/Sensitiser: ‘Innate’ means a surface is sensitive without further functionalisation.

**Table 3:** Summary of Langmuir–Freundlich (LF) parameters. Equation (2), for all fits to response characteristics shown in this work. c_1/2_ = 1/k. Limits-of-detection (LoDs) are determined in the Supplementary Section, S1.

Fig. No.	Analyte	Sensitiser	ΔV_sat_ [mV]	β	k [L/mol]	c_1/2_ [nM]	LoD
Figure 2	Cu^2+^	clinoptilolite	139.8 ± 3.9	0.807 ± 0.05	(6.95 ± 0.73) × 10^3^	(144 ± 15) × 10^3^	8.3 μM
Figure 3a	Hg^2+^	MTS9140+	203.2 ± 20.1	1.06 ± 0.11	(2.11 ± 0.48) × 10^6^	474 ± 108	17.7 nM
		MTS9200					
Figure 3b	Hg^2+^	MTS9140	122.9 ± 4.6	1.34 ± 0.12	(4.94 ± 0.44) × 10^6^	202 ± 18	73.7 nM
Figure 4	Hg^2+^	MTS9140+	103.6 ± 5	1.5 ± 0.14	(1.21 ± 0.1) × 10^7^	82.6 ± 6.8	17.1
		MTS9200					

## Data Availability

All experimental details are clarified in our experimental section. All discussion and conclusions are based on transfer characteristics that are shown as we recorded them in the manuscript. Data extracted from the transfers are plotted as clearly visible data points, and are fitted to mathematical models given in the manuscript. The resulting fit curves are shown in the same graphs; extracted fit parameters and their errors are tabulated in full. We therefore believe all data supporting our conclusions are fully reported in the present manuscript, and its open access online publication makes them permanently available to all interested parties.

## Supplementary Materials

The following supporting information can be downloaded at: https://www.mdpi.com/article/10.3390/s23177554/s1.
