# Supplementary material for: Bridged EGFET Design for the Rapid Screening of Sorbents as Sensitisers in Water-Pollution Sensors
Source: Sensors (Basel). 2023 Aug 31;23(17):7554. doi: 10.3390/s23177554 (PMC10490669; doi:10.3390/s23177554)
Supplement: Supplementary file 1 [file sensors-23-07554-s001.zip › sensors-2555769-supplementary.pdf]

Supplementary Material

S1: Evaluation of LoD

Limits-of-detection (LoDs) for LF response characteristics were determined by the method described in [17], section 2.4 / eq. 4: We re-plotted LF response characteristics in linearised form, $\Delta VCG(c)((kc)^{\beta}+1)$ vs. $(kc)^{\beta}$, and fitted a straight line of the form

$$\Delta V_{CG}(c)\ ((kc)^{\beta}+1) = m(kc)^{\beta} + b \qquad \text{(S1)}$$

As shown in Fig.s S1 to S4. Parameters m and b +/− Δb were evaluated by a linear fitting routine. Δb is expected to be larger or similar to b. LoDs then were calculated from the common '3 estimated standard errors' criterion [17]:

$$(kc_{LoD})^{\beta} = 3\ \Delta b/m \qquad \text{(S2)}$$

Fit parameters and resulting LoDs are summarised in table S1.

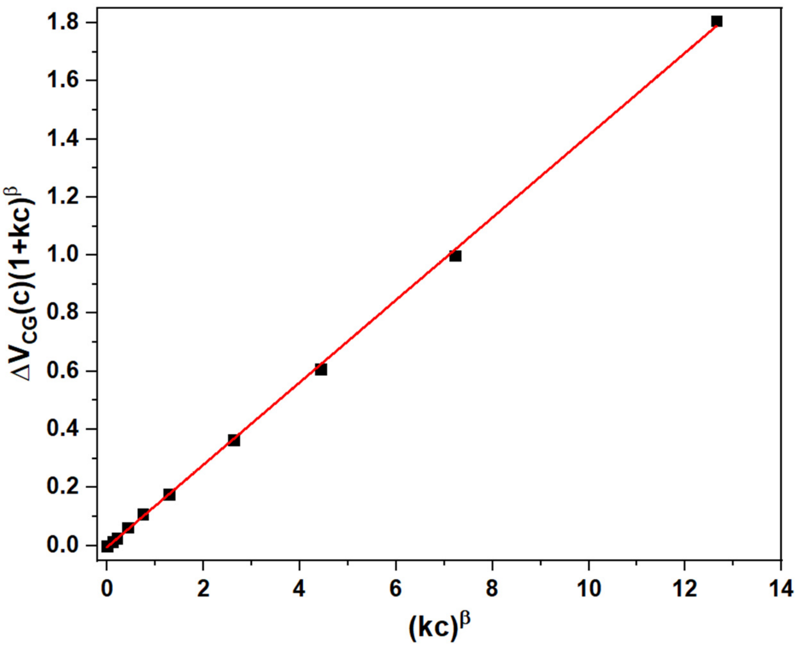

Figure S1: Fig. 2a replotted in the form $\Delta V_{CG}(c)((kc)^{\beta}+1)$ vs. $(kc)^{\beta}$

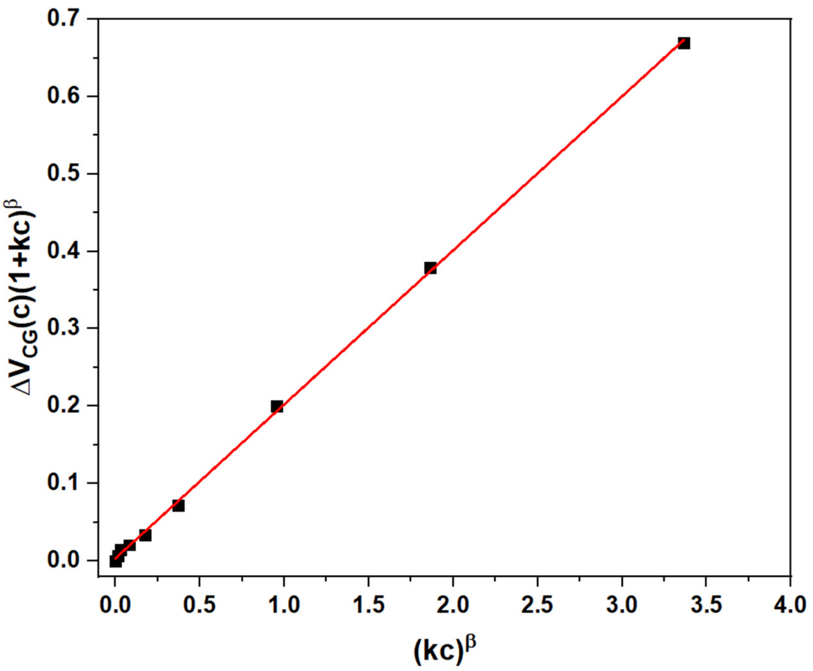

Figure S2: Fig. 3a replotted in the form $\Delta V_{CG}(c)((kc)^{\beta} + 1)$ vs. $(kc)^{\beta}$

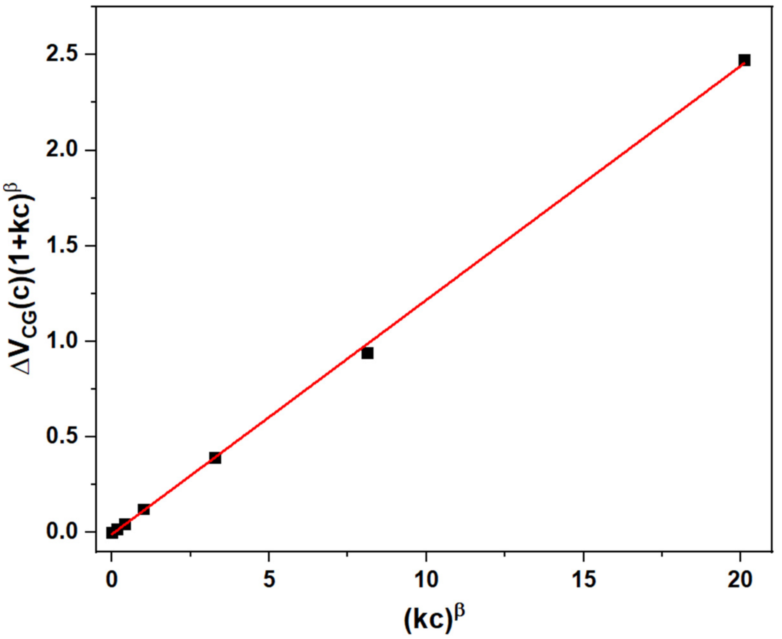

Figure S3: Fig. 3b replotted in the form $\Delta V_{CG}(c)((kc)^{\beta} + 1)$ vs. $(kc)^{\beta}$

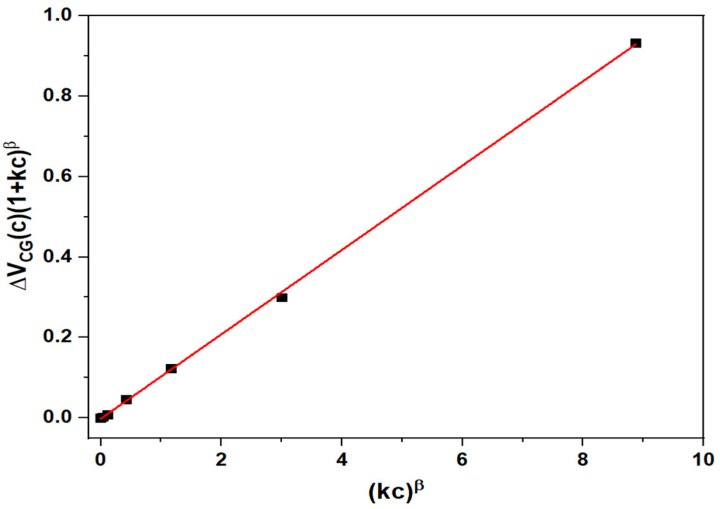

Fig S4: Fig. 4 replotted in the form $\Delta V_{CG}(c)((kc)^{\beta} + 1)$ vs. $(kc)^{\beta}$

Table S1

Figure	m [mV]	b [mV]	Δb [mV]	LoD
S1 / 2	141.8 ± 1	-4.6	4.8	8.3 µM
S2 / 3a	199.1 ± 1.5	3.4	2	17.7 nM
S3 / 3b	122.5 ± 1.3	-8.1	10.5	73.7 nM
S4 / 4	104.9 ± 0.8	-2	2.98	17.1 nM

Table S1: Fit parameters m, b, and Δb from Fig.s S1, S2, S3, S4, and evaluation of LoD with eq. S1 and k, β from table 3. Δb is larger or similar to b in all cases, which is consistent with the expectation b = 0.
